# Antiproliferative and antioxidant potentials of bioactive edible vegetable fraction of *Achyranthes ferruginea* Roxb. in cancer cell line

**DOI:** 10.1002/fsn3.2343

**Published:** 2021-06-01

**Authors:** A. S. M. Ali Reza, Md. Anwarul Haque, Joy Sarker, Mst. Samima Nasrin, Md. Mahbubur Rahman, Abu Montakim Tareq, Zidan Khan, Mamunur Rashid, Md. Golam Sadik, Toshifumi Tsukahara, AHM Khurshid Alam

**Affiliations:** ^1^ Department of Pharmacy International Islamic University Chittagong Chittagong Bangladesh; ^2^ Department of Pharmacy University of Rajshahi Rajshahi Bangladesh; ^3^ Doctoral Program in Biomedical Sciences Graduate School of Comprehensive Human Sciences University of Tsukuba Ibaraki Japan; ^4^ Department of Experimental Pathology Faculty of Medicine University of Tsukuba Ibaraki Japan; ^5^ Department of Pharmacy Varendra University Rajshahi Bangladesh; ^6^ School of Materials Science Japan Advanced Institute of Science and Technology Nomi City Japan

**Keywords:** *Achyranthes ferruginea*, antioxidants, cytotoxicity, free radical scavenging, GC‐MS, molecular docking

## Abstract

In the present study, the aerial parts of *Achyranthes ferruginea* underwent investigation of their in vitro antioxidant and free radical‐scavenging activities in cell‐free conditions, their phytoconstituents using gas chromatography‐mass spectrometry (GC‐MS), and their cytotoxic activity in HeLa cells. *A. ferruginea* was extracted with 80% methanol and successively fractionated with solvents to yield petroleum ether (PEF), chloroform (CHF), ethyl acetate (EAF), and aqueous (AQF) fractions. GC‐MS analysis revealed that CHF contained ten phytoconstituents, including different forms of octadecanoic acid methyl esters. The total antioxidant and ferric‐reducing antioxidant capacities of the extracts and the standard catechin (CA) were as follows: CA >CHF >PEF >CME (crude methanolic extract) >EAF >AQF, and CA >CHF >EAF >PEF >AQF >CME, respectively. CHF showed the highest DPPH‐free radical‐scavenging activity, with a median inhibitory concentration of 10.5 ± 0.28 µg/ml, which was slightly higher than that of the standard butylated hydroxytoluene (12.0 ± 0.09 µg/ml). In the hydroxyl radical‐scavenging assay, CHF showed identical scavenging activity (9.25 ± 0.73 µg/ml) when compared to CA (10.50 ± 1.06 µg/ml). Moreover, CHF showed strong cytotoxic activity (19.95 ± 1.18 µg/ml) in HeLa cells, which was alike to that of the standards vincristine sulfate and 5‐fluorouracil (15.84 ± 1.64 µg/ml and 12.59 ± 1.75 µg/ml, respectively). The in silico study revealed that identified compounds were significantly linked to the targets of various cancer cells and oxidative enzymes. However, online prediction by SwissADME, admetSAR, and PASS showed that it has drug‐like, nontoxic, and potential pharmacological actions.

## Graphical abstract

In this study, we found that the CHF of A. ferruginea has strong antioxidant and cytotoxic properties in different cell lines. GC‐MS quantitative phytochemical screening revealed that CHF contains 10 compounds which has known cytotoxic activity. Therefore, the obtained biological activity of A. ferruginea supports its use as a traditional medicine against different illnesses, and the plant can be considered a potential candidate for treating cancer.

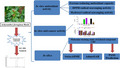

## 1 INTRODUCTION

Recently, a global study revealed the severity of cancer in both developed and developing countries (Dagenais et al., 2020). International Agency for Research on Cancer (IARC) estimates that 1 in 5 men and 1 in 6 women around the globe will affect by cancer in their lifetime, and of those, 1 in 8 men and 1 in 11 women will die (Bray et al., 2018). This is often because of lack of early detection methods and poor prognosis that accompanies diagnosis at an advanced rather than early stage. The GLOBOCAN 2018 database revealed 18.1 million new cancer and 9.6 million cancer deaths annually and will be over 13 million by 2030, particularly in the low‐ and middle‐income countries, including Bangladesh (Raychaudhuri & Mandal, 2012). Schutte reported that nearly 1 in 6 deaths in the world was due to cancer, making it the second leading cause of death (Afshin et al., 2019). Therefore, cancer is an alarming issue and there is an urgent need to develop awareness of the risk factors of cancer and to address the causes and control of this deadly disease.

Cancer is mainly caused by environmental and endogenous factors. About 90%–95% of cancers are due to environmental factors, and the remainder (5%–10%) are due to endogenous factors (Vogelstein & Kinzler, 2004). Humans are constantly exposed to environmental factors such as ultraviolet rays, drugs, and tobacco smoke, and to endogenous factors those derived from mitochondrial, microsomal, or peroxisomal activity in the electron transfer systems that cause oxidative stress (OS) (Siegel et al., 2016). In OS, active oxygen may be involved in carcinogenesis through the overexpression of oncogenes (e.g., *BCR*‐*ABL*, *BCL2*, *RAS*, *MYC*) and decreased expression of tumor‐suppressor genes (e.g., *BRCA1*, *BRCA2*, *RB*, *TP53*). The altered expression of these genes becomes more extensive if the cellular antioxidant defense system becomes weakened (Alam et al., 2021; Noda & Wakasugi, 2001; Siegel et al., 2016). Therefore, antioxidant‐enriched botanical supplements can confer protection by decreasing OS and inflammatory processes (Chow, 2010; Reza, Hossain, et al., 2018). In fact, in addition to their direct free radical‐scavenging capacity (Kumar & Pandey, 2015), flavonoids and their metabolites also impair free radical formation in the living body (Halliwell & Cross, 1994). The naturally occurring antioxidants curcumin and resveratrol have potential anticancer activity (Küpeli Akkol et al., 2020; Lee‐Chang et al., 2013; Pal et al., 2005). Raffoul et al. (2007) reported that phytoconstituent soy isoflavones (genistein, daidzein, glycitein) show anticancer activity.

Plants with medicinal properties are being used in health care since the dawn of civilization (Khan et al., 2020; Moni et al., 2021; Rahman et al., 2021; Sofowora et al., 2013). Globally, many studies have been conducted to check their effectiveness, with some of the results leading to plant‐based medicines (Goni et al., 2021; Hossen et al., 2021; Sofowora et al., 2013). The growing global burden of cancer requires a new treatment option, while herbal medicine presents a potential alternative to western medicine for cancer treatment (Ahmed et al., 2020; Bari et al., 2021; de Carvalho et al., 2020). A large number of plant species are now being used in cancer treatment or prevention. Accumulating evidence suggests that plant species have anticancer properties (Alam et al., 2016; Greenwell & Rahman, 2015; Islam et al., 2015; Martínez et al., 2020; Silva et al., 2020). Approximately 35,000 plants have been assessed for possible anticancer activities by the National Cancer Institute (NCI), and of these, about 3,000 plant species have shown reproductive anticancer activity (Desai et al., 2008; Iqbal et al., 2020; Vieira et al., 2020).

To reveal the anticancer prospective of Bangladeshi flora as botanical supplements, we selected as part of our ongoing research *Achyranthes ferruginea* (*A. ferruginea*), locally known as Roktoshirinchi. The plant refers to the Amaranthaceae family and is available throughout the south Asian countries. It is used as traditional medicine by the rural people of Bangladesh to cure various diseases. In native practice, the whole plant is used for treating shigellosis. It also has astringent and diuretic properties. In addition, the women of Bengal use it to induce abortion. Further, it is used in the management of constipation, dropsy, piles, boils, and skin erosion (Garnis et al., 2004). The leaves are considered emetic and are beneficial in hydrophobia and snake bites. Moreover, the extract of the plant is mixed with the extract of white rati for dropsy and gonorrhea. The extract of the whole plant is also effective against bronchial asthma. It has also been reported that the plant has contraceptive property in rats and hamsters (Watt, 1889). Previously, our research group reported several in vitro biological studies of the plant. Rahman *et al*. reported that the plant has antimicrobial activity against some pathogenic microorganisms and cytotoxic activity in brine shrimp nauplii (Rahman et al., 2007). Alam *et al*. reported that it has antidiarrheal activity (Alam et al., 2002). A subacute toxicity study revealed that the plant has no considerable toxic activity (Alam et al., 2006). All these reported in vitro biological data support the use of the plant as traditional medicine in different diseases. Moreover, we identified a compound, *N*‐*trans*‐feruloyl‐4‐methyldopamine, from the chloroform extract of *A. ferruginea* that showed potential antimicrobial effects (Hasan & Rashid, 2003). Although the plant has different in vitro cell‐free biological activities and is used in traditional medicine for several illnesses, there are no reported data on its antioxidant and antiproliferative activity.

Therefore, we designed the present study to investigate the in vitro antioxidant and free radical‐scavenging activities in cell‐free conditions, its phytoconstituents using gas chromatography/mass spectrometry (GC‐MS), and its cytotoxic activity in HeLa cervical cancer cells. The plant showed significant antioxidant, free radical‐scavenging, and cytotoxic activities due to the presence of different phytoconstituents, including different forms of octadecanoic acid methyl esters. Moreover, the computer‐aided model showed that the identified compounds could be a potent antioxidant and anticancer agent.

## 2 MATERIALS AND METHODS

### 2.1 Plant collection

The whole plant of *A. ferruginea* was collected from Meherchondi village, which is adjacent to Rajshahi University Campus, Bangladesh, in April 2018. An expert taxonomist from the University of Rajshahi, Department of Botany, identified the plant, and a voucher specimen representing this collection has been maintained at the Bangladesh National Herbarium (BNH) under the accession number DACB‐29533. Then, freshwater was used to remove dirt from the plant materials, and the plant was shade‐dried for several days, with occasional sun‐drying. The dried materials were ground into coarse powder by a grinding machine and were stored at room temperature (RT) for future use.

### 2.2 Extract preparation

**FIGURE 1:**
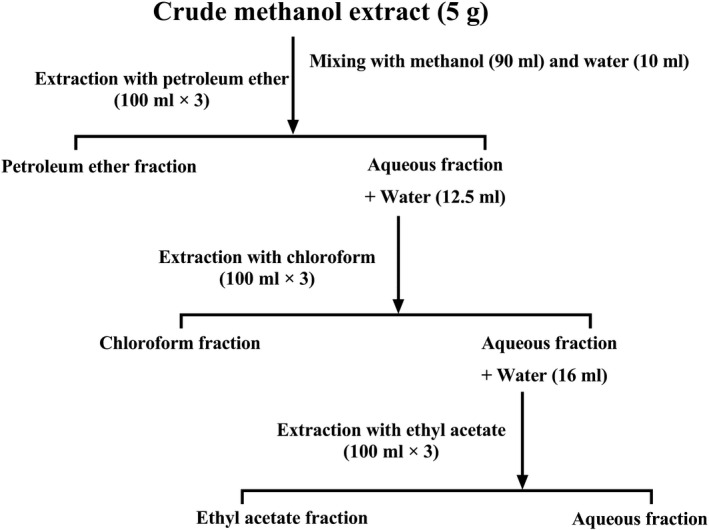
Schematic representation of the solvent‐solvent partitioning of methanol crude extract of *A. ferruginea*

Approximately 500 g powdered plant materials was soaked in about 1.5 L 80% methanol in an amber‐colored extraction bottle. The sealed bottles were stored for 7 days, with occasional shaking and stirring. Then, cotton filtration was performed first in the extraction, followed by filtration using Whatman No. 1 filter paper. Afterward, the extract was concentrated with a rotary evaporator (Bibby Sterilin Ltd., Staffordshire, UK) under reduced pressure at 50°C to yield 30 g crude methanolic extract (CME). The CME was then successively fractionated with petroleum ether, chloroform, ethyl acetate, and finally with water to obtain petroleum ether (PEF), chloroform (CHF), ethyl acetate (EAF), and aqueous (AQF) fractions (Figure 1) (Emran et al., 2015; Kupchan et al., 1973).

### 2.3 GC‐MS analysis of CHF

The bioactive compounds from CHF were analyzed using GC‐MS analysis followed by the method described earlier (Bulbul et al., 2019; Hossain et al., 2020).

### 2.4 Determination of total phenolics

The total phenolic contents of the extracts were determined using the modified Folin–Ciocalteu method as described by Wolfe and Islam *et al*. (Islam et al., 2013; Wolfe et al., 2003). Here, 500 µg/ml of the extract was mixed with 2 ml Folin–Ciocalteu reagent (previously diluted with water 1:10 v/v) and 2 ml (75 g/L) of sodium carbonate. The tubes were vortexed for 15 s and allowed to stand for 20 min at 25°C for color development. Absorbance was then measured at 760 nm UV‐spectrophotometer (Shimadzu). Total phenolic contents were expressed in terms of gallic acid equivalent, GAE (standard curve equation: y = 0.102x + 0.02687, R2 = 0.9999), and mg of GA/g of dry extract. Gallic acid (GA) stock solution (5 mg/1 ml methanol) was diluted in five concentrations (0.156–25.00 μg/ml) to obtain standard curve, where the range of absorbance values was 1.56–2.57. Moreover, there were five fractions of sample with a concentration of 0.5 mg/ml and the range of the absorbance values was 0.448–2.890. The experiment was repeated three times at each concentration.

### 2.5 Determination of total flavonoids

Total flavonoids were estimated using aluminum chloride (AlCl_3_) colorimetric assay as described by Zhishen and Islam *et al*. (Islam et al., 2013; Zhishen et al., 1999). To 0.5 ml of samples/standard, 150 μl of 5% sodium nitrate and 2.5 ml of distilled water were added. After 5 min, 0.3 ml of 10% AlCl_3_ was added. At 6 min, 1 ml of 0.001 M NaOH and 0.55 ml distilled water were added to the mixture and left at room temperature (RT) for 15 min. Absorbance of the mixtures was measured at 510 nm. Total flavonoid content was expressed in terms of catechin equivalent, CAE (standard curve equation: y = 0.005x + 0.012, R2 = 0.999), and mg of CA/g of dry extract. There were five concentrations ranged from 31.25 to 500.00 μg/ml of CA with the absorbance values ranged from 0.125 to 2.57, respectively. Samples of extract were evaluated at a final concentration of 0.5 mg/ml, and the range of the absorbance values was 0.034–0.688. The experiment was repeated three times at each concentration.

### 2.6 Determination of total antioxidants

The total antioxidant capacity of the extracts was determined with the phosphomolybdate method using CA as a standard as described by Prieto *et al*. (Prieto et al., 1999). The assay is based on the reduction in Mo (VI) to Mo (V) by samples and formation of green‐colored phosphate/Mo(V) complex at acidic pH. 0.5 ml of samples/standard at different concentrations (6.25–100 μg/ml) was mixed with 3 ml of reaction mixture containing 0.6 M sulfuric acid, 28 mM sodium phosphate, and 1% ammonium molybdate into the test tubes. The test tubes were incubated at 95°C for 10 min to complete the reaction. The absorbance was measured at 695 nm using a spectrophotometer against blank after cooling at RT. CA was used as standard. The absorbance values of the samples were 0.010–0.743 at the concentrations ranged from 6.25 to 100 μg/ml, respectively. The absorbance values of standard CA were 0.049–0.993 at the concentrations ranged from 6.25 to 100 μg/ml, respectively. A typical blank solution contained 3 ml of reaction mixture, the appropriate volume of the same solvent used for the samples/standard was incubated at 95°C for 10 min, and the absorbance was measured at 695 nm. Increased absorbance of the reaction mixture indicates increased total antioxidant capacity.

### 2.7 DPPH (2,2‐diphenyl‐1‐picrylhydrazyl) radical‐scavenging assay

The free radical‐scavenging ability of the extracts was tested using the DPPH radical‐scavenging assay as described by Blois (Blois, 1958) and Desmarchelier *et al*. (Desmarchelier et al., 1997). The hydrogen atom donating ability of the plant extracts was determined by the decolorization of methanol solution of 2,2‐diphenyl‐ 1‐picrylhydrazyl (DPPH). DPPH produces violet/purple color in methanol solution and fades to shades of yellow color in the presence of antioxidants. A solution of 0.1 mM DPPH (4 mg DPPH in 100 ml of 95% methanol) was prepared, and 2.4 ml of this solution was mixed with 1.6 ml of extracts in methanol at different concentration (6.25–100 μg/ml). The reaction mixture was vortexed thoroughly and left in the dark place at RT for 30 min. The absorbance of the mixture was measured spectrophotometrically at 517 nm. BHT was used as standard. Percentage DPPH radical‐scavenging activity was calculated by the following equation:

$$\%\;\text{DPPH radical}‐\text{scavenging activity}=\left[A_{0}‐A_{1}/A_{0}\right]\times 100$$

where A_0_ is the absorbance of the control, and A_1_ is the absorbance of the extracts/standard. Then, % of inhibition was plotted against concentration, and from the graph, IC_50_ was calculated. The range of the absorbance values of all the samples was 0.023–0.535 at the concentrations ranged from 6.25 to 100 μg/ml, respectively. The experiment was repeated three times at each concentration.

### 2.8 Ferric‐reducing antioxidant capacity

The ferric (Fe^3+^)‐reducing antioxidant capacity of the extracts/standard was evaluated by the method as described by Oyaizu (Oyaizu, 1986). The Fe^2+^ can be monitored by measuring the formation of Perl's Prussian blue at 700 nm. 0.25 ml samples/standard solution at different concentrations (6.25–100 μg/ml), 0.625 ml of potassium buffer (0.2 M), and 0.625 ml of 1% potassium ferricyanide [K_3_Fe (CN)_6_] solution were added into the test tubes. The reaction mixture was incubated for 20 min at 50°C to complete the reaction. Then, 0.625 ml of 10% TCA solution was added into the test tubes. The total mixture was centrifuged at 3,000 rpm for 10 min. After which, 1.8 ml supernatant was withdrawn from the test tubes and was mixed with 1.8 ml of distilled water and 0.36 ml of 0.1% ferric chloride (FeCl_3_) solution. The absorbance of the solution was measured at 700 nm using a spectrophotometer against blank. The range of the absorbance values of the samples was 0.008–0.981 at the concentrations ranged from 6.25 to 100 μg/ml, respectively. A typical blank solution contained the same solution mixture without plant extracts/standard was incubated under the same conditions, and the absorbance of the blank solution was measured at 700 nm. Increased absorbance of the reaction mixture indicates increased reducing capacity. The experiment was repeated three times at each concentration.

### 2.9 Hydroxyl radical‐scavenging assay

The hydroxyl radical‐scavenging activity (HRSA) of the extracts/standard was determined by the method described by Halliwell and Gutteridge (Halliwell, 1989). Hydroxyl radical was generated by the Fe3+‐ascorbate‐EDTA‐H_2_O_2_ system (Fenton reaction). The assay is based on the quantification of the 2‐deoxy‐D‐ribose degradation product, which forms a pink chromogen upon heating with TBA at low pH. The reaction mixture contained 0.8 ml of phosphate buffer solution (50 mmol/L, pH 7.4), 0.2 ml of extracts/standard at different concentration (6.25–100 μg/ml), 0.2 ml of EDTA (1.04 mmol L‐ 1), 0.2 ml of FeCl3 (1 mmol/L), and 0.2 ml of 2‐deoxy‐D‐ribose (28 mmol/L) was taken in the test tubes. The mixtures were kept in a water bath at 37°C, and the reaction was started by adding 0.2 ml of AA (2 mmol/L) and 0.2 ml of H_2_O_2_ (10 mmol/L). After incubation at 37°C for 1 hr, 1.5 ml of TBA (10 g L‐ 1) was added to the reaction mixture followed by 1.5 ml of HCl (25%). The mixture was heated at 100°C for 15 min and then cooled down with water. The absorbance of solution was measured at 532 nm with a spectrophotometer. The hydroxyl radical‐scavenging capacity was evaluated with the inhibition percentage of 2‐deoxy‐D‐ribose oxidation on hydroxyl radicals. The percentage of hydroxyl radical‐scavenging (%HRSA) activity was calculated according to the following formula:

The percentage (%) scavenging activity of HO• radicals was calculated from the following equation:

$$\%\;\text{HO}∙\text{radicals scavenging activity}=\left\{\left(\text{Ac}‐\text{As}\right)/\text{Ac}\right\}\times 100$$

where Ac=absorbance of the control, As=absorbance of the extract/standard. At the concentrations ranged from 6.25 to 100 μg/ml, the range of the absorbance values of the samples was 0.068–3.478, respectively. The experiment was repeated three times at each concentration.

### 2.10 Cell culture

HeLa cervical cancer cells were purchased from American Type Culture Collection. The cells were routinely cultured in Dulbecco's modified Eagle's medium (DMEM) supplemented with 10% fetal bovine serum in an atmosphere with 5% CO_2_ at 37°C. Exponential growth cells were used in all experiments.

### 2.11 Cell proliferation assay

Cell proliferation was assessed using the MTT assay (Islam et al., 2013). Briefly, 5 × 10^3^ cells were incubated in 96‐well plates in the presence of various concentrations of the fractions for 48 hr. At the end of the treatment, 20 μl MTT (5 mg/ml dissolved in PBS) was added to each well and incubated for an additional 4 hr at 37°C. The purple‐blue MTT formazan precipitate was dissolved in 200 μl of DMSO, the optical density was measured at 570 nm using microplate reader (Varioskan Flash 2.4.3., Thermo Fisher Scientific), and percentage of cell viability was calculated by using the following formula: OD of samples/OD of controlsX100.

### 2.12 In silico molecular docking

#### 2.12.1 Protein preparation

The 3D crystal structures of Caspase 3 (PDB: 5IAE) for HeLa (Luo et al., 2019; Maciag et al., 2016), structure of EGFR kinase domain for non‐small‐cell lung cancer cells (PDB: 2ITY) (Zhao et al., 2016), glutathione reductase (PDB: 3GRS) (Karplus & Schulz, 1987), and urate oxidase (PDB: 1R4U) (Retailleau et al., 2004) were downloaded in PDB format from Protein Data Bank (Berman et al., 2002). Then, the structures were prepared and refined followed by the method described by earlier Uddin *et al* (Uddin et al., 2018).

#### 2.12.2 Ligand preparation

Isolated compound from CHF fraction was retrieved from PubChem databases. The 3D structures for these were built by using LigPrep wizard in Maestro Schrödinger (v11.1) with an OPLS3 force field. Their ionization states were generated at pH 7.0 ± 2.0 using Epik 2.2 in Schrödinger Suite. Up to 32 possible stereoisomers per ligand were retained.

#### 2.12.3 Receptor grid generation

Receptor grids were calculated for the prepared proteins such that various ligand poses would bind within the predicted active site during docking (Bristy et al., 2020; Uddin et al., 2018). In Glide, grids were generated keeping the default parameters of van der Waals scaling factor 1.00 and charge cutoff 0.25 subjected to OPLS3 force field. A cubic box of specific dimensions centered on the centroid of the active site residues (Reference ligand active site) was generated for receptor. The bounding box was set to 14 Å × 14 Å × 14 Å for docking experiments.

#### 2.12.4 Glide standard precision (SP) ligand docking

Standard precision flexible ligand docking was carried out in Glide of Schrödinger‐Maestro (v11.1) (Friesner et al., 2004, 2006; Uddin et al., 2018) within which penalties were applied to noncis/trans amide bonds. Van der Waals scaling factor and partial charge cutoff were selected to be 0.80 and 0.15, respectively, for ligand atoms. Final scoring was performed on energy‐minimized poses and displayed as Glide score. The best docked pose with lowest Glide score value was recorded for each ligand.

#### 2.12.5 Determination of pharmacokinetic parameters by SwissADME

The pharmacokinetic properties of the isolated compound were evaluated using the SwissADME (http://www.swissadme.ch/) (Tareq et al., 2020). In the present study, an orally active drug should fulfill the drug‐likeness parameters (Lipinski et al., 1997) to demonstrate their pharmaceutical fidelity such as molecular weight of the compounds, lipophilicity (LogP), the number of hydrogen‐bond acceptors, the number of hydrogen‐bond donors, topological polar surface area (TPSA), and the number of rotatable bonds (nRB) based on Lipinski's and Veber's rules.

#### 2.12.6 Toxicological property prediction by AdmetSAR

Toxicological properties of the isolated compounds were determined using the admetSAR online tool (http://lmmd.ecust.edu.cn/admetsar1/predict/) sine toxicity is a prime concern during the development of new drugs. In the present study, Ames toxicity, carcinogenic properties, acute oral toxicity, and rat acute toxicity were predicted (Veber et al., 2002).

#### 2.12.7 In silico prediction of activity spectra for substances (PASS) study

The isolated compound from CHF fraction was examined for evaluating the anticancer, antiviral, free radical‐scavenging, lipid peroxidase inhibitor, and antioxidant activities by using PASS online (http://www.pharmaexpert.ru/passonline/).

### 2.13 Statistical analysis

Data are presented as the mean ±standard deviation (*SD*) from triplicate experiments. The data for significant differences between the test and control groups were described using one‐way analysis of variance (ANOVA), followed by Dunnett's test (GraphPad Prism data editor for Windows, version 6.0, GraphPad software Inc.). *p*‐values of <.05, <.01, and .001 were considered statistically significant.

## 3 RESULTS

### 3.1 GC‐MS analysis of CHF

**FIGURE 2:**
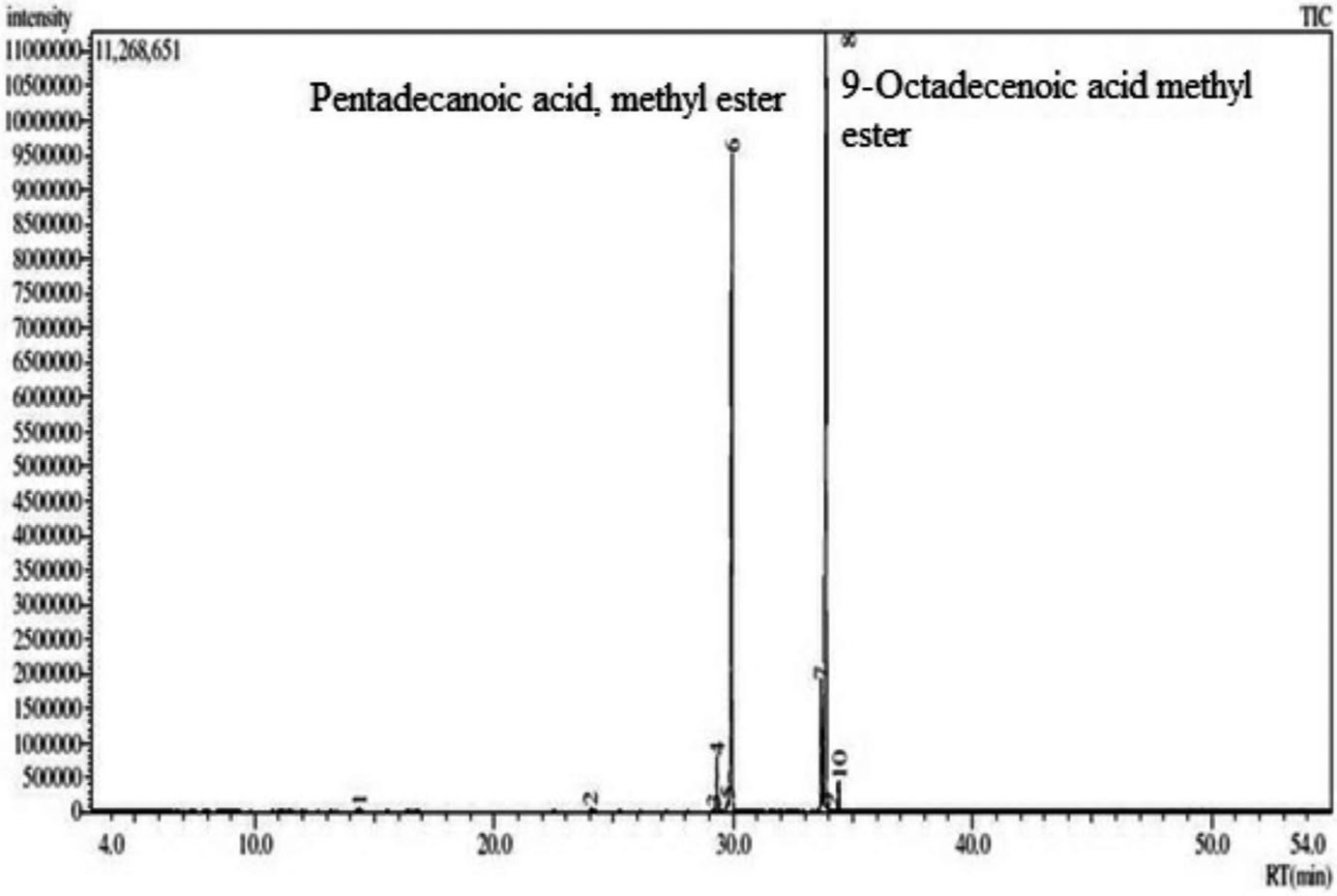
Gas chromatography‐mass spectrometry profile of CHF was obtained from GC‐MS with electron impact ionization (EI) method on a gas chromatograph (GC‐17A, Shimadzu Corporation) coupled to a mass spectrometer (GC‐MS TQ 8,040, Shimadzu Corporation)

The investigation of the presence of phytochemical compounds in CHF revealed several medicinally active compounds, which are summarized in Table 1. The quantitative phytochemical screening confirmed the presence of chemical constituents from the GC‐MS spectrum of CHF (Figure 2). A total of 10 compounds were confirmed using their mass spectra with that in the computer library. The main constituents were nonanoic acid, 9‐oxo‐methyl ester (0.104%), tridecanoic acid (0.240%), 7‐hexadecenoic acid, methyl ester, (Z)‐ (0.088%), 9‐hexadecenoic acid, methyl ester, (Z)‐ (1.788%), hexadecanoic acid methyl ester (0.834%), pentadecanoic acid methyl ester (65.130%), 9,12‐octadecadienoic acid methyl ester (4.219%), 9‐octadecenoic acid methyl ester, (E)‐ (25.794%), 11‐octadecenoic acid methyl ester (0.086%), and methyl stearate (1.717%).

**TABLE 1:** Compounds identified in the CHF fraction of *A*. *ferruginea* by GC‐MS

S.N.	Name of the compounds	RT	Peak area (%)	Nature
1.	Nonanoic acid, 9‐oxo‐, methyl ester	14.362	0.104	Fatty acid
2.	Tridecanoic acid, 12‐methyl‐, methyl ester	24.055	0.240	Fatty acid
3.	7‐Hexadecenoic acid, methyl ester, (Z)‐	29.200	0.088	Fatty acid
4.	9‐Hexadecenoic acid, methyl ester, (Z)‐	29.320	1.788	Fatty acid
5.	Hexadecanoic acid, methyl ester	29.750	0.834	Fatty acid methyl ester
6.	Pentadecanoic acid, methyl ester	29.949	65.130	Fatty acid methyl ester
7.	9,12‐Octadecadienoic acid, methyl ester	33.681	4.219	Fatty acid methyl ester
8.	9‐Octadecenoic acid, methyl ester, (E)‐	33.879	25.794	Fatty acid
9.	11‐Octadecenoic acid, methyl ester	33.963	0.086	Fatty acid
10.	Methyl stearate	34.407	1.717	Fatty acid methyl ester

### 3.2 Estimation of total phenolic content (TPC)

The TPC of CME, PEF, CHF, EAF, and AQF was 11.48 ± 0.045, 18.40 ± 0.015, 55.44 ± 0.010, 19.34 ± 0.035, and 8.38 ± 0.025 mg GAE/g dried extract, respectively, at a concentration of 500 µg/ml (Table 2). The TPC of the fractions was calculated using GA calibration curve. Table 2 shows that CHF and EAF had higher total phenolic content than the other fractions; hence, CHF and EAF might serve as a good source of antioxidants. The order of total phenolic compound content was as follows: CHF >EAF >PEF >CME >AQF.

**TABLE 2:** Polyphenol contents of CME and its various fractions at a concentration of 500 µg/ml

Polyphenols	CME	PEF	CHF	EAF	AQF
Phenolics^a^	11.48 ± 0.045^1^	18.40 ± 0.015	55.44 ± 0.010	19.34 ± 0.035	8.38 ± 0.025
Flavonoids^b^	88.41 ± 0.022	252.28 ± 0.011	277.48 ± 0.017	52.68 ± 0.021	19.61 ± 0.013

Note

NB: ^1^Each value is the average of three analyses ±standard deviation, a and b expressed in terms of GAE (mg of GA/g of dry extract, respectively).

### 3.3 Estimation of total flavonoid content (TFC)

The TFC of CME, PEF, CHF, EAF, and AQF was determined using the well‐known AlCl_3_ colorimetric method, using CA as the standard. The total flavonoid contents of CME, PEF, CHF, EAF, and AQF were 88.41 ± 0.022, 252.28 ± 0.011, 277.48 ± 0.017, 52.68 ± 0.021, and 19.61 ± 0.013 mg CAE/g dry sample, respectively (Table 2). CHF had the highest flavonoid content at the concentration of 500 µg/ml. The extracts’ phenolic and flavonoid contents reveal that *A. ferruginea* is rich in polyphenolic phytoconstituents, which might have good antioxidant activity.

### 3.4 Determination of total antioxidant capacity (TAC)

**FIGURE 3:**
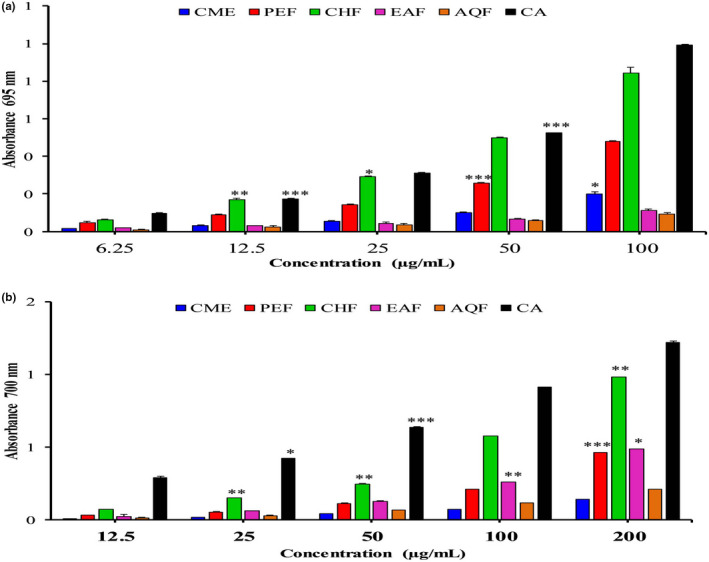
Determination of (a) total antioxidant capacity and (b) ferric‐reducing antioxidant capacity of CME and its various fractions (PEF, CHF, EAF, and AQF). Data expressed as mean ± *SD* (*n* = 3) for all tested dosages. Data were analyzed by one‐way ANOVA followed by Dunnett's test (GraphPad Prism data editor for Windows, version 6.0) for multiple comparisons. Values with (^*^p < .05, ^**^p < .01, ^***^p < .001) were considered significant. Where methanolic extract of *A. ferrugenea* (CME), petroleum ether (PEF), chloroform (CHF), ethyl acetate (EAF), and aqueous (AQF) fractions

The TAC of the *A. ferruginea* extracts was assessed using the spectrometric procedure, with CA as the standard. Figure 3a shows the TAC of CME with its four fractions, and of CA. Among the fractions, CHF showed the highest total antioxidant activity (absorbance, 0.843 ± 3.21) followed by PEF (absorbance, 0.479 ± 1.30), CME (absorbance, 0.200 ± 0.61), EAF (absorbance, 0.113 ± 0.06), and AQF (absorbance, 0.094 ± 0.77) at the concentration of 100 µg/ml. The absorbance of the standard CA was 0.993, which closely resembled that of CHF. Our results demonstrate that all *A. ferruginea* fractions revealed significant antioxidant activity (*p* < .05, *F* = 2.389). The TAC of the fractions and the CA were in the following order: CA >CHF >PEF >CME >EAF >AQF.

### 3.5 Ferric‐reducing antioxidant capacity (FRA)

The FRA of the *A. ferruginea* fractions was determined using the same method as above method and using CA as the standard. Figure 3b shows the absorbance values, from which it is clear that the CME and its various fractions have low reducing capacity (*p* < .05 and *F* = 26.94), although CHF had the highest absorbance value of 0.991 ± 0.98 at the concentration of 200 µg/ml. The order of reducing capacity of the fractions and the standard is as follows: CME <AQF <PEF <EAF <CHF <CA. The antioxidant (TAC and FRA) activity of *A. ferruginea* could be used to investigate whether it might have free radical‐scavenging potential.

### 3.6 DPPH radical‐scavenging activity

**FIGURE 4:**
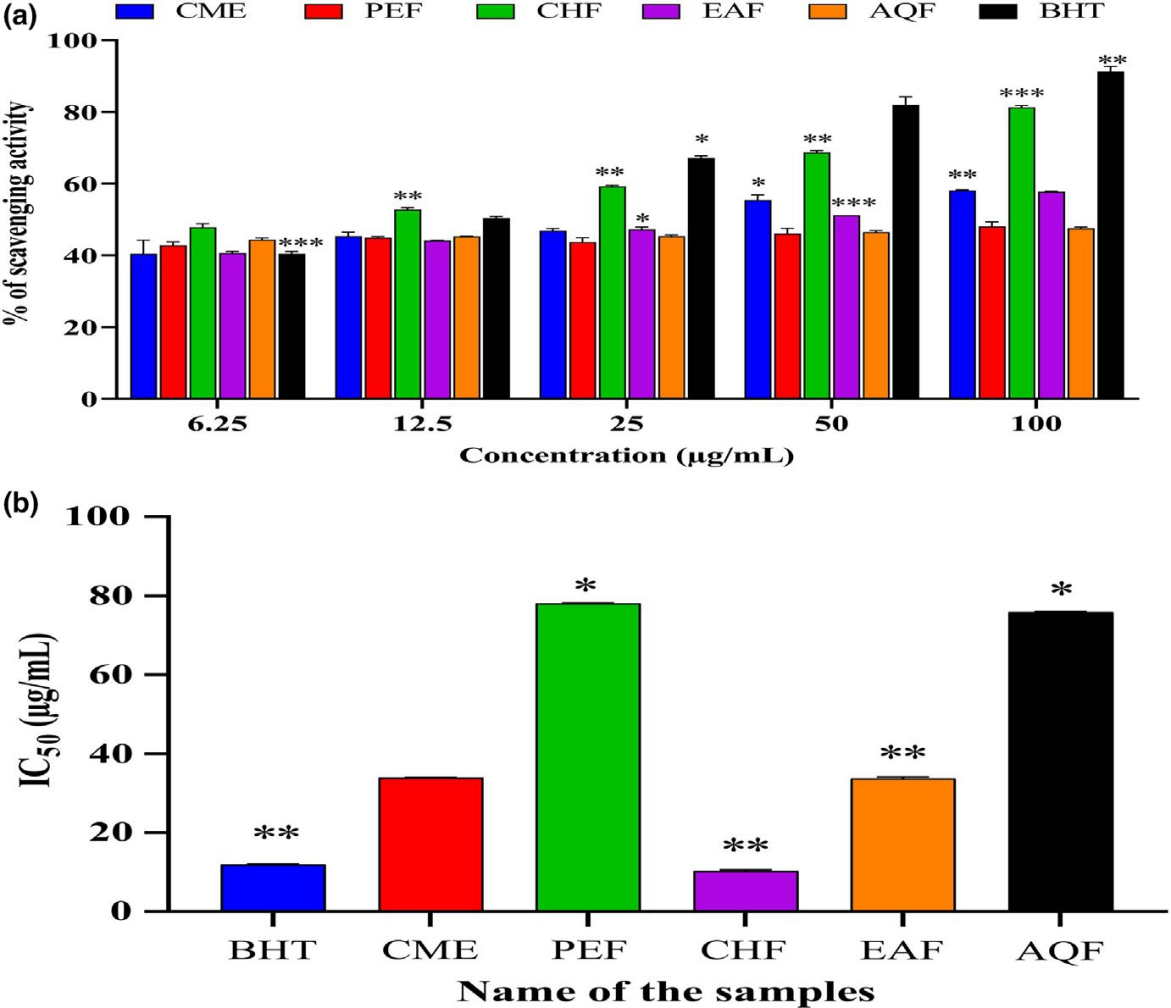
Determination of (a) DPPH radical‐scavenging activity and (b) IC_50_ of CME and its various fractions (PEF, CHF, EAF, and AQF). Data expressed as mean ± *SD* (*n* = 3) for all tested dosages. Data were analyzed by one‐way ANOVA followed by Dunnett's test (GraphPad Prism data editor for Windows, version 6.0) for multiple comparisons. Values with (^*^p < .05, ^**^p < .01, ^***^p < .001) were considered significant. Where methanolic extract of *A. ferrugenea* (CME), petroleum ether (PEF), chloroform (CHF), ethyl acetate (EAF), and aqueous (AQF) fractions

Figure 4a,b shows the results of the DPPH radical‐scavenging assay. CHF had the highest scavenging activity (91.63 ± 0.45%, *p* < .01, *F* = 3.384), with an IC_50_ of 10.5 ± 0.28 µg/ml, which was slightly higher than that of the standard BHT (IC_50_, 12.0 ± 0.09 µg/ml). PEF and AQF had the lowest scavenging activity. The CME, PEF, CHF, EAF, AQF, and BHT IC_50_ were 34.0 ± 0.07, 78.0 ± 0.071, 10.5 ± 0.28, 33.74 ± 0.38, 75.95 ± 0.07, and 12.0 ± 0.09 µg/ml, respectively. A lower IC_50_ indicates higher scavenging activity, suggesting that the *A. ferruginea* CHF has significant DPPH radical‐scavenging activity.

### 3.7 Hydroxyl radical‐scavenging activity

**FIGURE 5:**
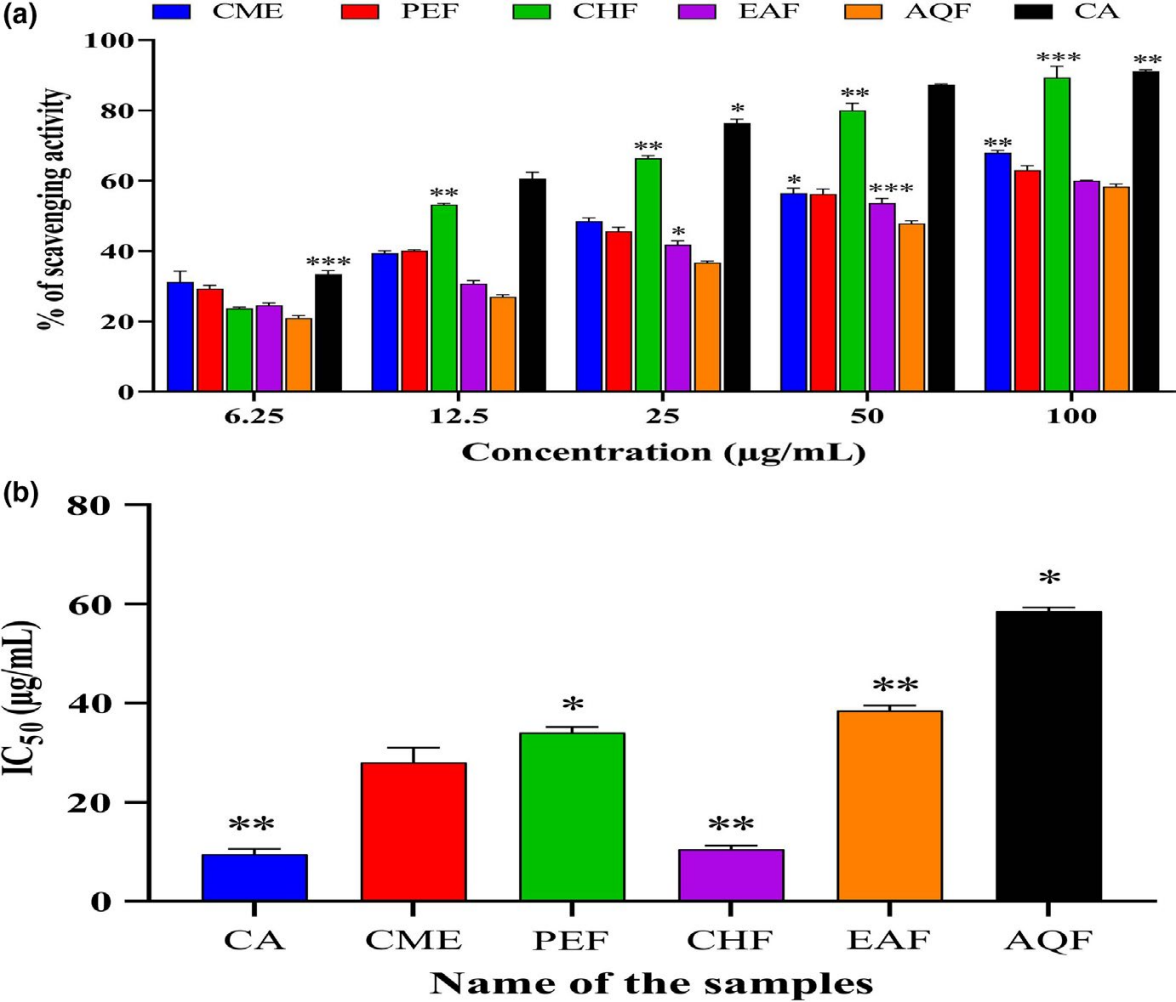
Determination of (a) hydroxyl radical‐scavenging activity and (b) IC_50_ of CME and its various fractions (PEF, CHF, EAF and AQF). Data expressed as mean ± *SD* (*n* = 3) for all tested dosages. Data were analyzed by one‐way ANOVA followed by Dunnett's test (GraphPad Prism data editor for Windows, version 6.0) for multiple comparisons. Values with (^*^p < .05, ^**^p < .01, ^***^p < .001) were considered significant. Where, methanolic extract of *A. ferrugenea* (CME), petroleum ether (PEF), chloroform (CHF), ethyl acetate (EAF), and aqueous (AQF) fractions

Figure 5a,b shows the HRSA results with the IC_50_ of CME and CA. CHF had the highest scavenging activity of all of the other fractions (*p* < .01, *F* = 1.756), being 89.32 ± 2.07%, with an IC_50_ of 9.25 ± 0.73 µg/ml, which was slightly higher than that of CA (IC_50_, 10.50 ± 1.06 µg/ml). The HRSA of the extracts and the standard CA were in the following order: CHF >CA >CME >PEF >EAF >AQF. Our results clearly demonstrate that *A. ferruginea* has potential radical‐scavenging activity that might have cytotoxic activity.

### 3.8 Determination of cytotoxic activity

**FIGURE 6:**
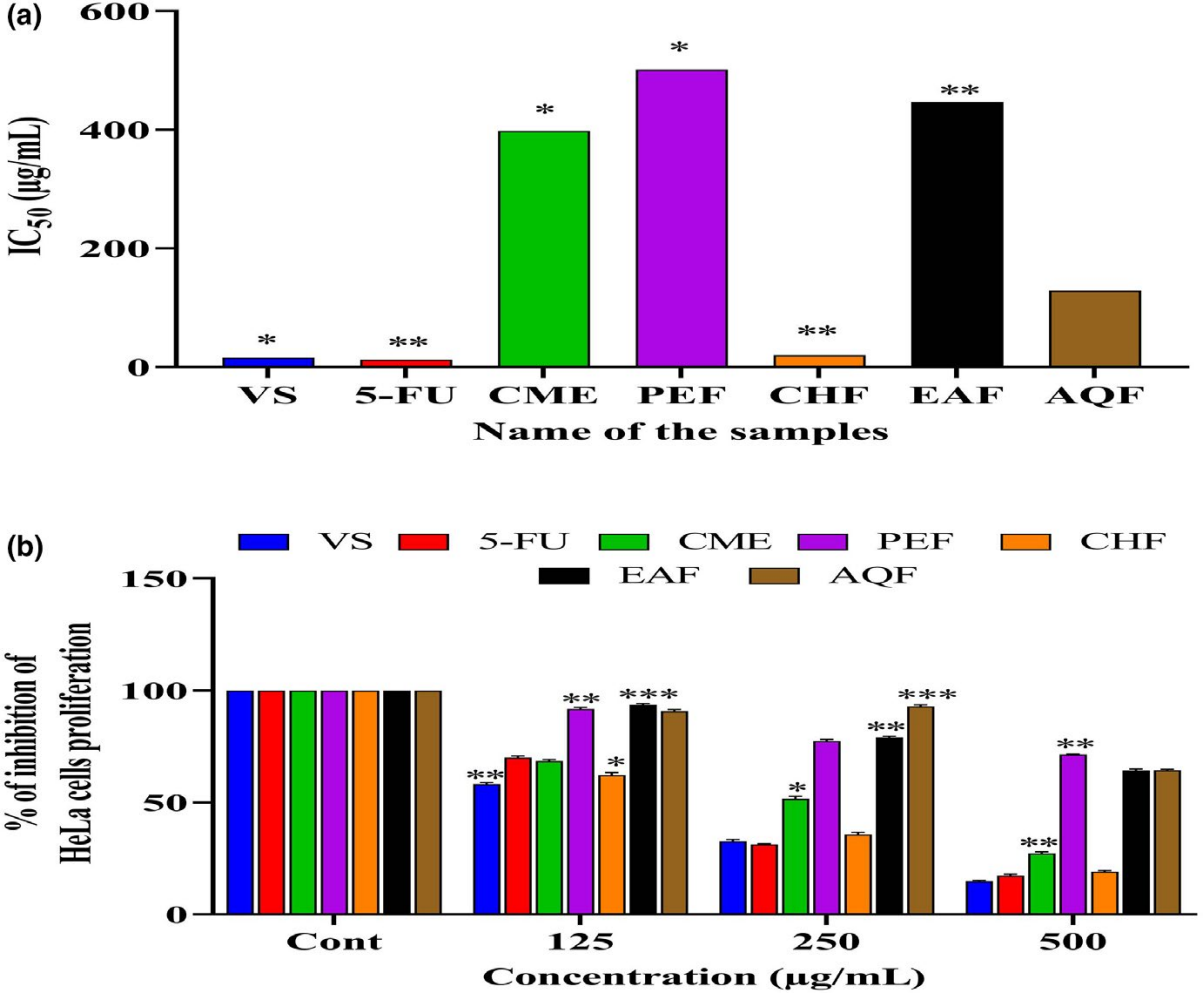
Determination of (a) IC_50_ of CME and its various fractions (PEF, CHF, EAF, and AQF) and (b) anticancer activity of CME and its various fractions (PEF, CHF, EAF, and AQF) using MTT. Data expressed as mean ± *SD* (*n* = 3) for all tested dosages. Data were analyzed by one‐way ANOVA followed by Dunnett's test (GraphPad Prism data editor for Windows, version 6.0, San Diego, CA) for multiple comparisons. Values with (^*^p < .05, ^**^p < .01, ^***^p < .001) were considered significant. Where methanolic extract of *A*. *ferrugenea* (CME), petroleum ether (PEF), chloroform (CHF), ethyl acetate (EAF), and aqueous (AQF) fractions

The cytotoxicity of the extracts was screened using the MTT cell proliferation assay. HeLa cells were treated with 125–500 μg/ml samples. Interestingly, AQF showed significant, dose‐dependent inhibition of HeLa cell proliferation (*p* < .01, *F* = 1.324), with an IC_50_ of 128.82 ± 1.80 µg/ml, whereas the standards VS and 5‐fluorouracil (5‐FU) had IC_50_ of 15.84 ± 1.64 µg/ml and 12.59 ± 1.75 µg/ml, respectively (Figure 6a). Then, we assessed the cytotoxicity of the fractionated extracts in HeLa cells, using MTT at the same concentration. CHF showed the highest cytotoxic activity, with an IC_50_ of 19.95 ± 1.18 µg/ml (*p* < .01, *F* = 1.324) (chi‐square value =40.78 and degree of freedom=12) which that was similar to that of the standards (Figure 6b), suggesting that the plant has anticancer activity. The antioxidant and cytotoxic activity of the plant is due to the presence of phytoconstituents, including different forms of octadecanoic acid methyl esters, which was confirmed by molecular docking in a computer‐aided model.

### 3.9 In silico molecular docking for antioxidant activity

**FIGURE 7:**
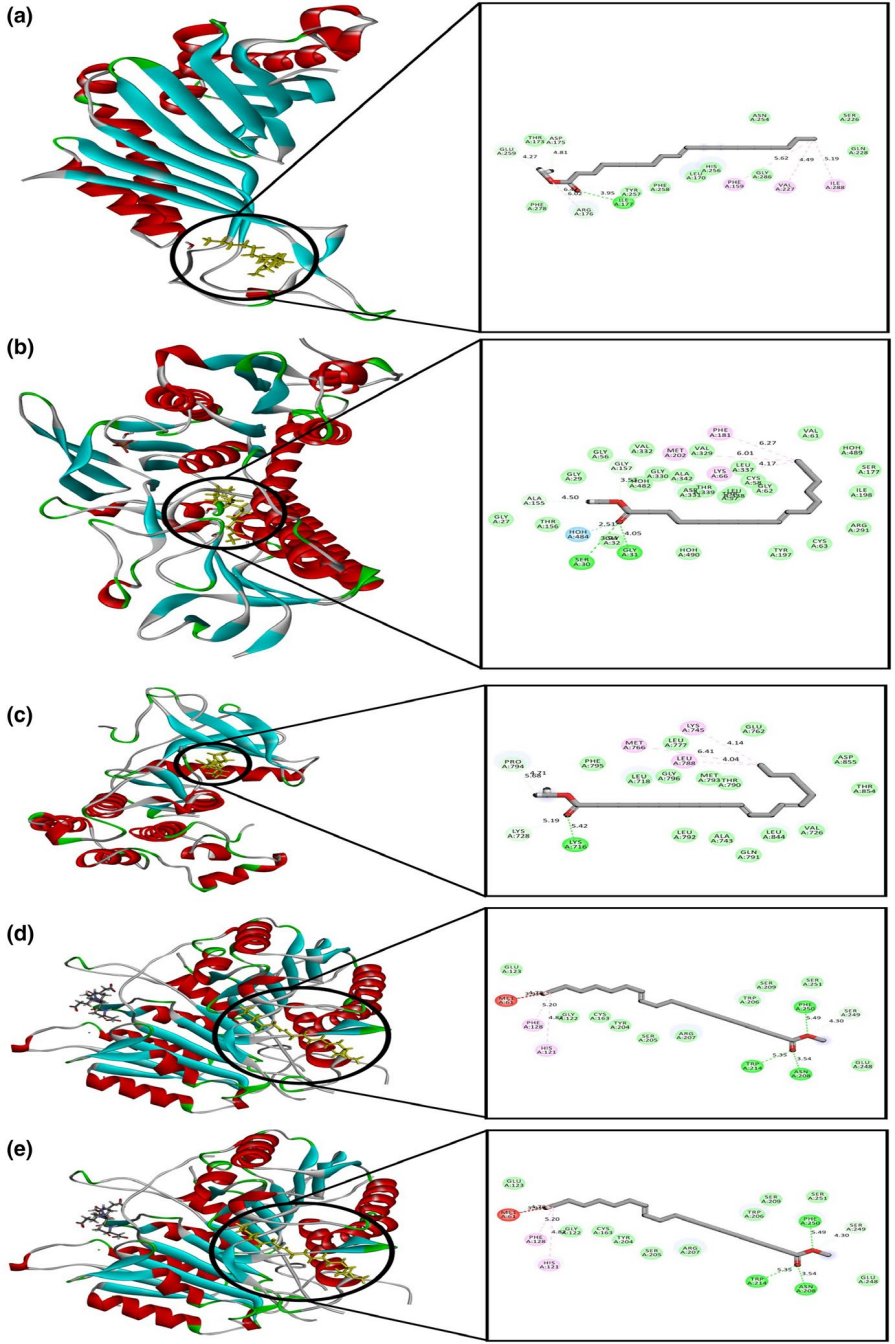
(a) 2D and 3D representation of best docking scores between: (a) urate oxidase (PDB: 1R4U) and 9‐octadecenoic acid methyl ester, (E)‐; (b) glutathione reductase (PDB: 3GRS) and 9‐Hexadecenoic acid, methyl ester for antioxidant activity. (b) 2D and 3D representation of best docking scores between: (c) non‐small‐cell lung cancer cells (PDB: 2ITY) and 9,12‐octadecadienoic acid, methyl ester; (d) Caspase 3 (PDB: 5IAE) and 11‐octadecenoic acid, methyl ester; (e) Caspase 3 (PDB: 5IAE) and Methyl stearate for anticancer activity

The results for molecular‐docking simulation study for ten selective compounds are delineated in Table 3. For antioxidant property, the compounds were docked against urate oxidase (PDB: 1R4U) and glutathione reductase (PDB: 3GRS), where 9‐octadecenoic acid methyl ester, (E)‐ exhibits the highest score (−0.401 kcal/mol) against urate oxidase receptor and 9‐hexadecenoic acid, methyl ester, (Z)‐ found to have highest docking score (−1.449 kcal/mol) against glutathione reductase receptor. The figure demonstrating the 2D and 3D conformation of ligand–protein complex for highest docking score is shown in Figure 7a. Along with that, the hydrogen bond and hydrophobic bond between the protein complex and the selective compounds are demonstrated in Tables 4 and 5.

**TABLE 3:** Molecular‐docking scores for the selected compounds

Compound	Docking score (kcal/mol)			
	1R4U	3GRS	2ITY	5IAE
7‐Hexadecenoic acid, methyl ester, (Z)‐	+0.652	−1.171	−0.126	−0.73
9,12‐Octadecadienoic acid, methyl ester	+0.691	−1.427	−1.284	−0.672
9‐Hexadecenoic acid, methyl ester, (Z)‐	+0.901	−1.449	−0.765	−0.195
9‐Octadecenoic acid methyl ester, (E)‐	+0.401	−0.655	−0.914	−0.564
11‐Octadecenoic acid, methyl ester	+1.022	−0.312	−0.497	−1.088
Hexadecanoic acid, methyl ester	+1.507	−0.447	−0.001	−0.029
Methyl stearate	+1.022	−0.312	−0.497	−1.088
Nonanoic acid, 9‐oxo‐, methyl ester	+0.446	−0.079	−0.516	+0.487
Pentadecanoic acid, methyl ester	+1.466	−0.878	−0.038	+0.35
Tridecanoic acid, 12‐methyl‐, methyl ester	+0.68	−1.381	−0.28	−0.614
Standard	Ascorbic acid(−4.655)	Ascorbic acid(−5.965)	5‐Fluorouracil (−5.218)	5‐Fluorouracil (−5.2)

**TABLE 4:** Interaction and bond distances of selective compounds and ascorbic acid with receptor urate oxidase (PDB: 1R4U)‐binding sites for antioxidant activity

Protein	Ligands	Hydrogen‐bond interactions		Hydrophobic interactions	
		Amino acid residue	Distance (Å)	Amino acid residue	Distance (Å)
1R4U	7‐Hexadecenoic acid, methyl ester, (Z)‐	LEU−170	4.17	PHE−159	3.99
		THR−169	3.99		
	9,12‐Octadecadienoic acid, methyl ester	HIS−256	3.61	ARG−176	4.76
				ILE−177	4.21
					4.41
				TYR−257	5.08
	9‐Hexadecenoic acid, methyl ester, (Z)‐	ASN−254	5.15	ILE−288	5.34
		HIS−256	3.71	PHE−159	5.47
		TYR−257	5.14	VAL−227	4.36
	9‐Octadecenoic acid methyl ester, (E)‐	ILE−177	3.95	ARG−176	6.02
				ARG−176	6.42
				GLU−259	4.27
				ASP−175	4.81
				PHE−159	5.62
				VAL−227	4.49
				ILE−288	5.19
	11‐Octadecenoic acid, methyl ester	HIS−256	3.69	TYR−257	4.45
		ASN−254	5.16		5.81
		TYR−257	0.12	ARG−176	4.87
				ILE−177	3.91
					3.67
	Hexadecanoic acid, methyl ester	ARG−176	6.46	ARG−176	5.55
				PHE−278	5.45
				ILE−177	3.78
				ILE−288	5.24
				GLN−228	4.31
				VAL−227	3.91
				ASN−254	4.49
				PHE−159	5.74
	Methyl stearate	HIS−256	3.69	TYR−257	5.81
		ASN−254	5.16		4.45
		TYR−257	0.12	ARG−176	4.87
				ILE−177	3.67
					3.91
	Nonanoic acid, 9‐oxo‐, methyl ester	ARG−176	6.91	SER−226	4.01
		VAL−227	3.86	LEU−163	4.35
				THR−168	4.08
	Pentadecanoic acid, methyl ester	ASN−254	4.90	HIS−256	3.92
		HIS−256	3.64	TYR−257	4.09
		TYR−257	4.67	LYS−255	6.34
				ILE−177	4.21
					4.40
				LEU−170	4.40
	Tridecanoic acid, 12‐methyl‐, methyl ester	TYR−257	5.48	TYR−257	6.33
				ILE−177	4.32
				PHE−278	6.24
				PHE−159	5.13
				VAL−227	4.05
				ILE−288	4.52
				ARG−176	4.95
	Ascorbic acid	HIS−256	4.03	TYR−257	6.50
		ILE−177	4.43		
			3.63		
		TYR−257	5.46		
		GLU−259	4.18		

**TABLE 5:** Interaction and bond distances of selective compounds and ascorbic acid with receptor glutathione reductase (PDB: 3GRS)‐binding sites for antioxidant activity

Proteins	Ligands	Hydrogen‐bond interactions		Hydrophobic interactions	
		Amino acid residue	Distance (Å)	Amino acid residue	Distance (Å)
3GRS	7‐Hexadecenoic acid, methyl ester, (Z)‐	THR−57	3.76	GLY−2.95	2.95
				GLY−56	4.22
				ALA−155	5.03
				MET−202	5.24
				PHE−181	6.35
				ILE−198	4.87
				LYS−66	4.55
	9,12‐Octadecadienoic acid, methyl ester	THR−339	3.71	PRO−340	4.98
				LEU−338	4.00
					5.16
				CYS−63	4.79
				TYR−197	6.62
					2.63
					5.93
	9‐Hexadecenoic acid, methyl ester, (Z)‐	SER−30	3.94	HOH−484	2.51
		GLY−31	4.04	ALA−155	4.50
				GLY−157	3.53
				LEU−337	4.17
				VAL−329	6.01
				PHE−181	6.27
	9‐Octadecenoic acid methyl ester, (E)‐	THR−339	3.97	THR−339	3.68
				CYS−63	5.98
				LEU−33	5.25
				TYR−114	4.42
	11‐Octadecenoic acid, methyl ester	THR−339	4.20	LEU−338	4.12
				CYS−63	5.80
				VAL−59	5.39
				TYR−114	5.45
				LEU−33	5.61
	Hexadecanoic acid, methyl ester	THR−339	3.86	LEU−338	3.98
				LEU−337	5.53
				CYS−63	6.07
				TYR−114	5.22
				ILE−33	4.45
	Methyl stearate	THR−339	4.20	LEU−338	4.12
				CYS−63	5.80
				VAL−59	5.39
				TYR−114	5.45
				LEU−33	5.61
	Nonanoic acid, 9‐oxo‐, methyl ester	THR−339	4.04	CYS−55	4.30
				LEU−338	5.55
				CYS−63	4.78
				HOH−484	2.61
				GLY−31	3.79
	Pentadecanoic acid, methyl ester	THR−57	3.63	THR−156	4.77
				ALA−155	4.65
				HOH−484	3.26
				LYS−66	4.92
				ILE−198	4.92
				PHE−181	6.07
				MET−5.06	5.06
	Tridecanoic acid, 12‐methyl‐, methyl ester	ASP−331	3.52	HOH−482	2.74
				ALA−155	4.06
				GLY−157	3.72
				GLY−330	4.11
				ILE−198	4.37
				MET−202	4.65
				LYS−66	4.77
				PHE−181	6.21
	Ascorbic acid	GLU−50	4.60	HOH−490	3.11
			4.15		
			4.93		
		ALA−155	4.10		
		THR−57	3.67		

### 3.10 In silico molecular docking for anticancer activity

To determine anticancer activity of the selective compounds, two protein structures, namely EGFR kinase domain for non‐small‐cell lung cancer cells (PDB: 2ITY) and Caspase 3 (PDB: 5IAE) for HeLa, were used. Among the ten compounds, 9,12‐octadecadienoic acid, methyl ester possessed the highest docking score (−1.284 kcal/mol) against for EGFR kinase domain. In case of Caspase 3, 11‐octadecenoic acid, methyl ester and methyl stearate both exhibit highest docking score (−1.088 kcal/mol). The docking score for each selective compound against these two proteins is given in Table 3. The structure of interaction for the highest docking score is given in Figure 7b. Also the hydrogen bond and hydrophobic interaction elaborating the amino acid residue are given in Tables 6 and 7.

**TABLE 6:** Interaction and bond distances of selective compounds and 5‐fluorouracil with receptor non‐small‐cell lung cancer cells (PDB: 2ITY) binding sites for anticancer activity

Proteins	Ligands	Hydrogen‐bond interactions			Hydrophobic interactions		
		Amino acid residue		Distance (Å)	Amino acid residue	Distance (Å)	
2ITY	7‐Hexadecenoic acid, methyl ester, (Z)‐		LYS−716	5.53	LYS−728		5.46
					MET−766		6.11
					LYS−745		4.57
					LEU−788		4.27
	9,12‐Octadecadienoic acid, methyl ester		LYS−716	5.42	LYS−728		5.19
					PRO−794		5.68
					PRO−794		4.71
					LEU−788		4.04
					MET−766		6.41
					LYS−745		4.14
	9‐Hexadecenoic acid, methyl ester, (Z)‐		LYS−716		LYS−745		6.43
							4.09
					LEU−788		3.62
	9‐Octadecenoic acid methyl ester, (E)‐		LYS−745	3.97	ASP−855		3.75
							4.52
	11‐Octadecenoic acid, methyl ester		LYS−716	5.50	THR−790		4.35
					ALA−743		3.84
	Hexadecanoic acid, methyl ester		LYS−716	5.42	MET−766		5.97
					LEU−788		3.47
	Methyl stearate		LYS−716	5.50	ALA−743		3.84
					LYS−745		4.35
	Nonanoic acid, 9‐oxo‐, methyl ester		MET−793	4.24	MET−793		3.81
			LYS−425	4.29	LEU−792		4.15
					LYS−745		3.80
					MET−788		6.12
					LEU−788		2.96
							3.77
					LRU−788		4.19
					LYS−745		4.48
	Pentadecanoic acid, methyl ester		LYS−716	5.37	PRO−794		5.87
							5.30
					PHE−795		5.33
					MET−766		5.96
					LEU−788		3.47
	Tridecanoic acid, 12‐methyl‐, methyl ester				LEU−788		2.64
							3.65
							4.22
					MET−766		5.82
							5.39
					LYS−745		2.85
					LEU−792		4.93
					LEU−718		4.92
							5.14
	5‐fluorouracil		ASP−855	3.60	LYS−745		4.42
			LYS−745	4.30	VAL−726		5.89

**TABLE 7:** Interaction and bond distances of selective compounds and 5‐fluorouracil with receptor Caspase 3 (PDB: 5IAE) for HeLa‐binding sites for anticancer activity

Proteins	Ligands	Hydrogen‐bond interactions		Hydrophobic interactions	
		Amino acid residue	Distance (Å)	Amino acid residue	Distance (Å)
5IAE	7‐Hexadecenoic acid, methyl ester, (Z)‐	PHE−250	5.40	GLU−248	5.24
		ASN−208	4.17	TRP−214	5.33
		TRP—214	5.24	ARG−207	4.03
				TRP−206	4.06
				LEU−168	5.56
				PHE−256	5.26
				TYR−204	3.63
	9,12‐Octadecadienoic acid, methyl ester	PHE−250	5.50	SER−249	4.44
		TRP−214	5.49	CYS−163	4.20
		ASN−208	3.49		
	9‐Hexadecenoic acid, methyl ester, (Z)‐	ASN−208	3.71	GLU−248	5.34
		TRP−214	5.28		5.46
		PHE−250	5.35	HIS−121	4.14
				CYS−153	4.26
	9‐Octadecenoic acid methyl ester, (E)‐	PHE−250	5.39	SER−249	4.29
		TRP−214	5.34	GLU−248	5.81
		ASN−208	3.69	CYS−163	4.67
	11‐Octadecenoic acid, methyl ester	PHE−250	5.49	MET−61	4.78
		TRP−214	5.35		3.91
		ASN−208	3.54	PHE−128	5.20
				HIS−121	4.82
				SER−249	4.30
	Hexadecanoic acid, methyl ester	TRP−214	5.02	TRP−214	4.76
		ASN−208	3.74	PHE−247	4.52
				PHE−256	5.12
				TYR−204	3.50
				LEU−168	5.20
				TRP−206	4.17
	Methyl stearate	PHE−250	5.49	MET−61	4.78
		TRP−214	5.35		3.91
		ASN−208	3.54	PHE−128	5.20
				HIS−121	4.82
				SER−249	4.30
	Nonanoic acid, 9‐oxo‐, methyl ester	PHE−250	5.53	SER−249	4.25
		ASN−208	3.61		
		TRP−214	5.17		
		ARG−207	5.16		
	Pentadecanoic acid, methyl ester	ASN−208	3.88	TRP−214	4.65
		TRP−214	5.12	PHE−247	4.44
				TYR−204	2.91
				TRP−206	4.28
				PHE−256	5.70
	Tridecanoic acid, 12‐methyl‐, methyl ester	PHE−250	5.85	TRP−214	5.45
		ASN−208	4.07	GLU−248	5.32
		TRP−214	5.18	PHE−256	5.14
				LEU−168	5.27
				TYR−204	3.48
				TYR−208	4.74
				TRP−206	4.21
					3.46
	5‐fluorouracil	SER−120	4.67	CYS−163	5.84
		GLN−161	3.89	HIS−121	3.51
		ARG−64	4.89	ARG−207	3.78

### 3.11 Determination of pharmacokinetic parameters and prediction of toxicological properties

The absorption, distribution, metabolism, and excretion (ADME) properties of the selected compounds were evaluated on the basis of Lipinski and Veber rule. The data for each of the parameters were extrapolated from swissADME online server which has been shown in Table 8. It was found that none of the compound has violated more than one parameter of the standard basis. This indication suggested that all of the compounds possess drug‐like property and auspicious oral bioavailability. Also, the toxicity profile for each of the selective compounds is evaluated through AdmetSAR online server and has given in Table 9. It depicted that all of the compounds are non‐Ames toxic and have little or insignificant rat acute toxicity which indicates that all of the compounds are free from toxicity.

**TABLE 8:** Physicochemical properties of the isolated compound from CHF for good oral bioavailability

Compound	Lipinski Rules				Lipinski's Violations	Veber Rules	
	MW	HBA	HBD	Log P		nRB	TPSA
	<500	<10	<5	≤5	≤1	≤10	≤140
Nonanoic acid, 9‐oxo‐, methyl ester	186.25	3	0	2.08	0	9	43.37
Tridecanoic acid, 12‐methyl‐, methyl ester	242.40	2	0	4.75	0	12	26.30
7‐Hexadecenoic acid, methyl ester, (Z)‐	268.43	2	0	5.22	1	14	26.30
9‐Hexadecenoic acid, methyl ester, (Z)‐	268.43	2	0	5.26	1	14	26.30
Hexadecanoic acid, methyl ester	270.45	2	9	5.54	2	15	26.30
Pentadecanoic acid, methyl ester	256.42	2	9	5.21	2	14	26.30
9,12‐Octadecadienoic acid, methyl ester	294.47	2	0	5.69	1	15	26.30
9‐Octadecenoic acid methyl ester, (E)‐	269.49	2	0	5.95	1	16	26.30
11‐Octadecenoic acid, methyl ester	296.49	2	9	5.95	2	16	26.30
Methyl stearate	298.50	2	0	6.24	1	17	26.30

Abbreviations: HBA, hydrogen‐bond acceptor; HBD, hydrogen‐bond donor; Log P, lipophilicity; MW, molecular weight (g/mol); nRB: number of rotatable bond; TPSA: topological polar surface area.

**TABLE 9:** Toxicological properties identified compounds from CHF fraction

Compound	Parameters			
	Ames toxicity	Carcinogens	Acute oral	Rat acute toxicity
Nonanoic acid, 9‐oxo‐, methyl ester	NAT	NC	III	2.1465
Tridecanoic acid, 12‐methyl‐, methyl ester	NAT	NC	III	1.5702
7‐Hexadecenoic acid, methyl ester, (Z)‐	NAT	Carcinogens	III	1.7357
9‐Hexadecenoic acid, methyl ester, (Z)‐	NAT	Carcinogens	III	1.7357
Hexadecanoic acid, methyl ester	NAT	Carcinogens	III	1.4915
Pentadecanoic acid, methyl ester	NAT	Carcinogens	III	1.4915
9,12‐Octadecadienoic acid, methyl ester	NAT	Carcinogens	III	1.7357
9‐Octadecenoic acid methyl ester, (E)‐	NAT	Carcinogens	III	1.7357
11‐Octadecenoic acid, methyl ester	NAT	Carcinogens	III	1.7357
Methyl stearate	NAT	Carcinogens	III	1.4915

Abbreviations: NAT, non‐Ames toxic; NC, noncarcinogenic; NR, nonrequired. Category‐III (500 mg/kg > LD_50_< 5000mg/kg).

### 3.12 PASS prediction study by PASS online

We investigated the PASS of the isolated compounds using the structure‐based Pass Online biological activity prediction program. If the value of probable activity (Pa) is higher than that of probable inactivity (Pi), the compound is considered a compound with pharmacological potential (Table 10). The Pa value of the isolated compounds indicates its prominent biological activity including free radical‐scavenging, antiviral, and anticancer activities.

**TABLE 10:** Biological activities predicted for identified compounds by PASS online

Name	Characteristics	Biological properties predicted by pass online	*p*_a_	*p*_i_
Nonanoic acid, 9‐oxo‐, methyl ester	Antioxidant	Free radical scavenger	.211	.068
		Peroxidase substrate	.292	.043
		Reductant	.274	.096
	Antiviral	Antiviral	.186	.111
		Antiprotozoal (Toxoplasma)	.289	.019
		Antiviral (Influenza)	.287	.098
		Antiviral (Rhinovirus)	.497	.025
		Antiviral (Picornavirus)	.405	.105
		Antiviral (Adenovirus)	.308	.083
		Antiviral (CMV)	.328	.017
		Antiviral (Herpes)	.291	.098
		Antiviral (Influenza A)	.239	.119
		Antiviral (Poxvirus)	.272	.077
	Anticancer	Antineoplastic, Alkylator	.226	.015
		Antineoplastic (cervical cancer)	.146	.063
		Antineoplastic (endocrine cancer)	.214	.038
		Antineoplastic (bone cancer)	.225	.080
		Antineoplastic (thyroid cancer)	.222	.019
		Antineoplastic antimetabolite	.194	.034
		Antineoplastic (solid tumors)	.250	.139
		Antineoplastic (Bladder cancer)	.153	.124
Tridecanoic acid, 12‐methyl‐, methyl ester	Antioxidant	Antioxidant	.243	.038
		Free radical scavenger	.312	.028
		Reductant	.384	.055
		Lipid peroxidase inhibitor	.302	.063
	Antiviral	Antiviral	.234	.070
		Antiviral (Influenza)	.388	.050
		Antiviral (Rhinovirus)	.595	.007
		Antiviral (Picornavirus)	.482	.058
		Antiviral (Adenovirus)	.404	.028
		Antiviral (CMV)	.379	.006
		Antiviral (Herpes)	.394	.038
		Antiviral (Hepatitis B)	.243	.054
		Antiviral (Poxvirus)	.253	.093
		Antiviral (HIV)	.142	.075
		Antiviral (Hepatitis)	.124	.064
		Antiviral (Hepatitis C)	.106	.051
		Antiviral (Parainfluenza)	.056	.017
	Anticancer	Antineoplastic (non‐Hodgkin's lymphoma)	.478	.047
		Antineoplastic (endocrine cancer)	.252	.025
		Antineoplastic (bone cancer)	.255	.027
		Antineoplastic (thyroid cancer)	.209	.025
		Antineoplastic, alkylator	.171	.027
		Antineoplastic antimetabolite	.159	.044
		Antineoplastic (liver cancer)	.186	.103
		Antineoplastic (solid tumors)	.227	.170
		Antineoplastic antibiotic	.078	.063
7‐Hexadecenoic acid, methyl ester, (Z)‐	Antioxidant	Antioxidant	.269	.030
		Free radical scavenger	.380	.019
		Peroxidase substrate	.548	.007
		Reductant	.594	.012
		Lipid peroxidase inhibitor	.326	.056
	Antiviral	Antiviral	.165	.141
		Antiviral (Influenza)	.492	.024
		Antiviral (Influenza A)	.208	.200
		Antiviral (Rhinovirus)	.639	.004
		Antiviral (Picornavirus)	.497	.052
		Antiviral (Adenovirus)	.387	.035
		Antiviral (CMV)	.488	.003
		Antiviral (Herpes)	.415	.029
		Antiviral (Herpesvirus 3, Human)	.013	.006
		Antiviral (Hepatitis B)	.181	.114
		Antiviral (Poxvirus)	.279	.071
		Antiviral (Parainfluenza)	.049	.023
	Anticancer	Antineoplastic (non‐Hodgkin's lymphoma)	.425	.086
		Antineoplastic (endocrine cancer)	.163	.102
		Antineoplastic (thyroid cancer)	.197	.034
		Antineoplastic, alkylator	.189	.021
		Antineoplastic antimetabolite	.148	.050
		Antineoplastic (liver cancer)	.273	.030
		Antineoplastic (solid tumors)	.244	.145
		Antineoplastic antibiotic	.090	.054
		Antineoplastic (lymphoma)	.137	.125
9‐Hexadecenoic acid, methyl ester, (Z)‐	Antioxidant	Antioxidant	.269	.030
		Free radical scavenger	.380	.019
		Peroxidase substrate	.548	.007
		Reductant	.594	.012
		Lipid peroxidase inhibitor	.326	.056
	Antiviral	Antiviral	.165	.141
		Antiviral (Influenza)	.492	.024
		Antiviral (Influenza A)	.208	.200
		Antiviral (Rhinovirus)	.639	.004
		Antiviral (Picornavirus)	.497	.052
		Antiviral (Adenovirus)	.387	.035
		Antiviral (CMV)	.488	.003
		Antiviral (Herpes)	.415	.029
		Antiviral (Herpesvirus 3, Human)	.013	.006
		Antiviral (Hepatitis B)	.181	.114
		Antiviral (Parainfluenza)	.049	.023
	Anticancer	Antineoplastic (non‐Hodgkin's lymphoma)	.425	.086
		Antineoplastic (endocrine cancer)	.163	.102
		Antineoplastic (thyroid cancer)	.197	.034
		Antineoplastic, alkylator	.189	.021
		Antineoplastic antimetabolite	.148	.050
		Antineoplastic (liver cancer)	.273	.030
		Antineoplastic (solid tumors)	.244	.145
		Antineoplastic antibiotic	.090	.054
		Antineoplastic (lymphoma)	.137	.125
Hexadecanoic acid, methyl ester	Antioxidant	Antioxidant	.210	.050
		Free radical scavenger	.332	.025
		Peroxidase substrate	.424	.017
		Reductant	.523	.020
		Lipid peroxidase inhibitor	.292	.067
	Antiviral	Antiviral	.176	.125
		Antiviral (Influenza)	.417	.041
		Antiviral (Influenza A)	.227	.145
		Antiviral (Rhinovirus)	.616	.005
		Antiviral (Picornavirus)	.554	.031
		Antiviral (Adenovirus)	.425	.020
		Antiviral (CMV)	.438	.004
		Antiviral (Herpes)	.392	.039
		Antiviral (Herpesvirus 3, Human)	.012	.008
		Antiviral (Hepatitis B)	.219	.070
		Antiviral (Poxvirus)	.352	.039
		Antiviral (Parainfluenza)	.072	.009
	Anticancer	Antineoplastic (non‐Hodgkin's lymphoma)	.409	.099
		Antineoplastic (endocrine cancer)	.214	.038
		Antineoplastic (bone cancer)	.220	.094
		Antineoplastic (thyroid cancer)	.222	.019
		Antineoplastic, alkylator	.218	.016
		Antineoplastic antimetabolite	.205	.032
		Antineoplastic (liver cancer)	.215	.069
		Antineoplastic (solid tumors)	.238	.154
		Antineoplastic (sarcoma)	.165	.117
		Antineoplastic (bladder cancer)	.156	.117
		Antineoplastic (insulinoma)	.012	.009
Pentadecanoic acid, methyl ester	Antioxidant	Antioxidant	.210	.050
		Free radical scavenger	.332	.025
		Peroxidase substrate	.424	.017
		Reductant	.523	.020
		Lipid peroxidase inhibitor	.292	.067
	Antiviral	Antiviral	.176	.125
		Antiviral (Influenza)	.417	.041
		Antiviral (Influenza A)	.227	.145
		Antiviral (Rhinovirus)	.616	.005
		Antiviral (Picornavirus)	.554	.031
		Antiviral (Adenovirus)	.425	.020
		Antiviral (CMV)	.438	.004
		Antiviral (Herpes)	.392	.039
		Antiviral (Herpesvirus 3, Human)	.012	.008
		Antiviral (Hepatitis B)	.219	.070
		Antiviral (Poxvirus)	.352	.039
		Antiviral (Parainfluenza)	.072	.009
	Anticancer	Antineoplastic (non‐Hodgkin's lymphoma)	.409	.099
		Antineoplastic (endocrine cancer)	.214	.038
		Antineoplastic (bone cancer)	.220	.094
		Antineoplastic (thyroid cancer)	.222	.019
		Antineoplastic, alkylator	.218	.016
		Antineoplastic antimetabolite	.205	.032
		Antineoplastic (liver cancer)	.215	.069
		Antineoplastic (solid tumors)	.238	.154
		Antineoplastic (sarcoma)	.165	.117
		Antineoplastic (bladder cancer)	.156	.117
		Antineoplastic (insulinoma)	.012	.009
9,12‐Octadecadienoic acid, methyl ester	Antioxidant	Antioxidant	.296	.024
		Free radical scavenger	.332	.025
		Peroxidase substrate	.591	.005
		Reductant	.637	.009
		Lipid peroxidase inhibitor	.354	.048
	Antiviral	Antiviral (Influenza)	.441	.035
		Antiviral (Rhinovirus)	.627	.005
		Antiviral (Picornavirus)	.458	.071
		Antiviral (Adenovirus)	.362	.047
		Antiviral (CMV)	.466	.003
		Antiviral (Herpes)	.401	.035
		Antiviral (Herpesvirus 3, Human)	.011	.009
		Antiviral (Hepatitis B)	.168	.139
		Antiviral (Poxvirus)	.257	.089
		Antiviral (Parainfluenza)	.043	.030
	Anticancer	Antineoplastic (non‐Hodgkin's lymphoma)	.389	.118
		Antineoplastic (thyroid cancer)	.189	.042
		Antineoplastic, alkylator	.167	.028
		Antineoplastic antimetabolite	.132	.060
		Antineoplastic (liver cancer)	.291	.023
		Antineoplastic (solid tumors)	.231	.164
		Antineoplastic (lymphoma)	.181	.069
		Antineoplastic antibiotic	.089	.055
9‐Octadecenoic acid methyl ester, (E)‐	Antioxidant	Antioxidant	.269	.030
		Free radical scavenger	.380	.019
		Peroxidase substrate	.548	.007
		Reductant	.594	.012
		Lipid peroxidase inhibitor	.326	.056
	Antiviral	Antiviral	.165	.141
		Antiviral (Influenza)	.492	.024
		Antiviral (Influenza A)	.208	.200
		Antiviral (Rhinovirus)	.639	.004
		Antiviral (Picornavirus)	.497	.052
		Antiviral (Adenovirus)	.387	.035
		Antiviral (CMV)	.488	.003
		Antiviral (Herpes)	.415	.029
		Antiviral (Herpesvirus 3, Human)	.013	.006
		Antiviral (Hepatitis B)	.181	.114
		Antiviral (Poxvirus)	.279	.071
		Antiviral (Parainfluenza)	.049	.023
	Anticancer	Antineoplastic (non‐Hodgkin's lymphoma)	.425	.086
		Antineoplastic (endocrine cancer)	.163	.102
		Antineoplastic (thyroid cancer)	.197	.034
		Antineoplastic, alkylator	.189	.021
		Antineoplastic antimetabolite	.148	.050
		Antineoplastic (liver cancer)	.273	.030
		Antineoplastic (solid tumors)	.244	.145
		Antineoplastic (lymphoma)	.137	.125
		Antineoplastic antibiotic	.090	.054
11‐Octadecenoic acid, methyl ester	Antioxidant	Antioxidant	.269	.030
		Free radical scavenger	.380	.019
		Peroxidase substrate	.548	.007
		Reductant	.594	.012
		Lipid peroxidase inhibitor	.326	.056
	Antiviral	Antiviral	.165	.141
		Antiviral (Influenza)	.492	.024
		Antiviral (Influenza A)	.208	.200
		Antiviral (Rhinovirus)	.639	.004
		Antiviral (Picornavirus)	.497	.052
		Antiviral (Adenovirus)	.387	.035
		Antiviral (CMV)	.488	.003
		Antiviral (Herpes)	.415	.029
		Antiviral (Herpesvirus 3, Human)	.013	.006
		Antiviral (Hepatitis B)	.181	.114
		Antiviral (Poxvirus)	.279	.071
		Antiviral (Parainfluenza)	.049	.023
	Anticancer	Antineoplastic (non‐Hodgkin's lymphoma)	.425	.086
		Antineoplastic (endocrine cancer)	.163	.102
		Antineoplastic (thyroid cancer)	.197	.034
		Antineoplastic, alkylator	.189	.021
		Antineoplastic antimetabolite	.148	.050
		Antineoplastic (liver cancer)	.273	.030
		Antineoplastic (solid tumors)	.244	.145
		Antineoplastic (lymphoma)	.137	.125
		Antineoplastic antibiotic	.090	.054
		Antineoplastic antibiotic	.639	.004
Methyl stearate	Antioxidant	Antioxidant	.210	.050
		Free radical scavenger	.332	.025
		Peroxidase substrate	.424	.017
		Reductant	.523	.020
		Lipid peroxidase inhibitor	.292	.067
	Antiviral	Antiviral	.176	.125
		Antiviral (Influenza)	.417	.041
		Antiviral (Influenza A)	.227	.145
		Antiviral (Rhinovirus)	.616	.005
		Antiviral (Picornavirus)	.554	.031
		Antiviral (Adenovirus)	.425	.020
		Antiviral (CMV)	.438	.004
		Antiviral (Herpes)	.392	.039
		Antiviral (Herpesvirus 3, Human)	.012	.008
		Antiviral (Hepatitis B)	.219	.070
		Antiviral (Poxvirus)	.352	.039
		Antiviral (Parainfluenza)	.072	.009
	Anticancer	Antineoplastic (non‐Hodgkin's lymphoma)	.409	.099
		Antineoplastic (endocrine cancer)	.214	.038
		Antineoplastic (thyroid cancer)	.222	.019
		Antineoplastic, alkylator	.218	.016
		Antineoplastic antimetabolite	.205	.032
		Antineoplastic (liver cancer)	.215	.069
		Antineoplastic (solid tumors)	.238	.154
		Antineoplastic (insulinoma)	.012	.009
		Antineoplastic (bone cancer)	.220	.094
		Antineoplastic (sarcoma)	.165	.117
		Antineoplastic (Bladder cancer)	.156	.117

Note

Where Pa =probable activity; Pi =probable inactivity.

## 4 DISCUSSION

Over the last century, plant secondary metabolites and their derivatives have played a pivotal role in combating cancer. According to the National Cancer Institute (NCI), around 35,000 plants and 114,000 plant samples from 20 different countries were examined for exploring novel anticancer drugs (Shoeb, 2006). In 2000, researchers observed that 14 of the top 35 cancer‐fighting drugs were obtained from natural products and their derivatives (Shoeb, 2006). Accumulating evidence suggests that plants are rich in polyphenolic compounds, including phenols, flavonoids, proanthocyanidins, and tannins (Pandey & Rizvi, 2009). Fotsis et al., (1997) reported that flavonoids showed antimutagenic and antimalignant activity (Fotsis et al., 1997). Moreover, phenolic compounds have antitumor, antimutagenic, and chemoprotective activity (Wagner et al., 1986). Many research studies have revealed that regular dietary intake of natural antioxidants reduces cardiovascular (CVS) disease and cancer mortality (Ansari et al., 2017; Mobarak et al., 2018; Prasad et al., 2010). Dietary polyphenols from plants or plant extracts have relatively higher radical‐scavenging activity than reference antioxidants, including vitamins E and C, and GA (Fresco et al., 2006; Nasrin et al., 2018). In our study, all fractions showed moderate to strong antioxidant activity, with the greatest amount of total phenolic and flavonoid compounds (Table 2). CHF had the maximum content of phenols and flavonoids and also demonstrated the strongest antiradical activity. Based on these observations, we summarized that the extracts’ radical‐scavenging nature might depend on their polyphenolic content, that is, phenolics and flavonoids. It is evident that total phenols and flavonoids are potential antioxidants and free radical scavengers (Adnan et al., 2020a; M. R. Islam et al., 2014; Reza, Hossain, et al., 2018). Moreover, polyphenolic compounds have a wide spectrum of chemical and biological activity, including antioxidant and anticancer properties (S. Islam et al., 2013; Reza et al., 2018).

Gas chromatography‐mass spectrometry analysis of CHF of *A. ferruginea* identified 10 compounds (Figure 2 and Table 1). Octadecanoic acid is a saturated fatty acid found in comparatively high concentrations in some plants. Octadecanoic acid is the primary metabolite present in plants, which forms glycerol esters. It has also been shown that octadecanoic acid has antitumor activity in mouse models and is selectively cytotoxic for MOLT‐4 leukemia cancer cells due to its interaction with DNA topoisomerase I and its ability to induce apoptosis (Chujo et al., 2003). In addition, hexadecanoic acid methyl ester has antioxidant, nematicide, pesticide, anti‐inflammatory, and antiandrogenic properties (Kim et al., 2014). Here, CHF contained a prominent amount of hexadecanoic acid methyl ester (0.834%), 9,12‐octadecadienoic acid methyl ester (4.219%), 9‐octadecenoic acid methyl ester (25.794%), and 11‐octadecenoic acid methyl ester (0.086%). GC‐MS analysis of CHF identified methyl stearate. Furthermore, stearic acid (stearate) is comparatively abundant in our dietary foods and possesses several biological activities, including breast cancer development and neoplastic progression (Evans et al., 2009). As CHF contains these bioactive compounds, it might play a significant role in the exploration of new anticancer natural compounds. Furthermore, the cytotoxic activity was ascertained by the presence of an active plant metabolite named *N*‐*trans*‐feruloyl‐4‐methyldopamine (Hasan & Rashid, 2003) and its antioxidant and cytotoxic activity was confirmed by molecular docking using a computer‐aided model.

The TAC was measured by the Mo (VI) to Mo (V) reduction capacity and the subsequent formation of a green phosphate/Mo (V) complex at an acidic pH. All fractions exhibited significant TAC (*p* < .05, *F* = 2.389), and the activity was concentration‐dependent. Among the fractions, CHF had the maximum TAC, followed by PEF, EAF, and AQF (Figure 3a). The highest antioxidant activity of CHF was due to the presence of polyphenolics. Previously, many researchers have proven that the TAC of citrus was due to the presence of phenolics, flavonoids, and AA (Jayaprakasha et al., 2008). The FRA capacity is considered as another prominent indicator of antiradical activity (Oliveira et al., 2008). The mechanism of FRA capacity is associated with the presence of a reductant that donates a hydrogen atom after ROS breakdown. In the current study, CHF showed the highest FRA capacity through its reduction in the Fe^3+^‐ferricyanide complex into the ferrous form, which was monitored through the blue‐green complex at 700 nm. The order of the reducing power of the fractions and the standard was as follows: CME <AQF <PEF <EAF<CHF <CA. The significant reducing power of CHF (*p* < .05 and *F* = 26.94) was possibly due to the presence of phenolic constituents, which might act as electron donors (Figure 3b).

Antioxidant activity is assessed based on the ability to reduce the stable DPPH radical (Desmarchelier et al., 1997). A stable diamagnetic molecule is formed by the DPPH radical by accepting an electron or hydrogen radical, which is followed by the solution changing color from blue to yellow. This color‐changing technique is a widely accepted in vitro method because of its simplicity, stability, and reproducibility (Reddy et al., 2008). All *A. ferruginea* fractions showed significant DPPH scavenging activity (*p* < .01, *F* = 3.384) (Figure 4a). The order of the IC_50_ of CME, PEF, CHF, EAF, AQF, and the standard BHT was as follows: CHF >BHT >CME >EAF >PEF >AQF. CHF had a significant IC_50_ value (*p* < .01) that was almost identical to that of BHT (Figure 4b). Duan *et al*. (20,007) reported that phenolics and flavonoids can reduce DPPH radicals by donating hydrogen ions (Duan et al., 2007). Our results are consistent with that of the data published previously (Islam et al., 2013; Khan et al., 2013; Reza, Nasrin, et al., 2018). As *A. ferruginea* has potent antioxidant capacity, it can donate hydrogen ions; consequently, it can be considered a radical scavenger. Hydroxyl radicals are responsible for biological damage through the activation of lipid peroxidation. The hydroxyl radicals attack the fatty acid side chains of the membrane phospholipids and add a double bond to DNA bases (Halliwell, 1991). The modification of the DNA bases may initiate different cellular process malfunctions, which are characterized by the primary pace of carcinogenesis (Pandey et al., 2012). Our results demonstrate that most of the fractions showed significant potential for scavenging hydroxyl radicals (*p* < .01, *F* = 1.756) (Figure 5a,b). CHF had higher scavenging activity than the other fractions, and the scavenging activity of CHF was similar to that of the standard CA. Moreover, CHF could protect against cancer by minimizing the chemical modification of genetic materials. We note that *A. ferruginea* has antioxidant potential and the capacity to combat oxidative damage because of its reducing power capacity as well as radical‐scavenging activity.

ROS initiates OS, which harmfully changes numerous cellular structures, such as membranes, lipids, proteins, lipoproteins, and DNA (Reza, Hossain, et al., 2018). OS occurs when there is imbalance between ROS and the loss of cell potential for eradicating ROS. For example, excess hydroxyl radicals and peroxynitrite can cause lipid peroxidation, injuring cell membranes and lipoproteins (Ayala et al., 2014) and consequently leading to the formation of malondialdehyde (MDA) and conjugated diene compounds, which are cytotoxic and mutagenic (Ayala et al., 2014). As stated earlier, cancer is a complicated process mediated by both cellular and molecular modifications. Endogenous and/or exogenous stimuli can play crucial roles in this alteration process. Cancer can be stimulated by chromosomal irregularities and oncogene initiation determined by OS (Reuter et al., 2010). A by‐product termed hydrolyzed DNA is formed by DNA oxidation (Valko et al., 2004), and this by‐product initiates transcriptomic changes, and genes are mutated. Furthermore, ROS are responsible for modifying DNA–protein cross‐links, base and sugar lesions, strand breaks, and base‐free sites (Pizzino et al., 2017). Antioxidants are a class of compounds that mop up ROS and prevent cancer. In the present study, we analyzed the cytotoxic activity of *A. ferruginea* on HeLa cells. CME and CHF showed strong dose‐dependent inhibition (*p* < .01, *F* = 1.324) of HeLa cell proliferation when compared with the standards VS and 5‐FU (Figure 6a,b).

Computer‐aided analysis revealed a good picture of antioxidant and anticancer compounds from *A. ferruginea*. Molecular‐docking study allows accurate prediction of ligand and receptor interaction as well as binding energy, to have a good picture of antioxidant and anticancer activities of the identified compounds. This research conducted a grid‐based in silico analysis of this active compound with the active site of HeLa cells, non‐small‐cell lung cancer cells, glutathione reductase, and urate oxidase. The interaction between the compound and the active sites was assessed with docking analysis in Schrodinger Suite v 11.1.

To identify potential lead molecule from CHF for antioxidant and anticancer activity, we performed grid‐based in silico analysis of this active compound with the active site of HeLa cells, non‐small‐cell lung cancer cells, glutathione reductase, and urate oxidase. The interaction between the compound and the active sites was assessed with docking analysis in Schrodinger Suite v 11.1. Among the compounds 9‐octadecenoic acid methyl ester, (E)‐ exhibits the highest score (−0.401 kcal/mol) against urate oxidase receptor and 9‐hexadecenoic acid methyl ester, (Z)‐ found to have highest docking score (−1.449 kcal/mol) against glutathione reductase receptor. Earlier researcher showed that 9‐octadecenoic acid methyl ester and 9‐hexadecenoic acid methyl ester have antioxidant activity (Shaheed et al., 2019). It has been proven that the negative and low value of the free energy of binding demonstrates a strong favorable bond. In addition, the molecular docking determined that the anticancer activity of identified compounds with HeLa cells and non–small lung cancer cells displayed a negative and low value of free energy of binding, demonstrating a strong favorable bond. Among the compounds, 9,12‐octadecadienoic acid methyl ester possessed the highest docking score (−1.284 kcal/mol) against for EGFR kinase domain. In case of Caspase 3, 11‐octadecenoic acid, methyl ester and methyl stearate both exhibit highest docking score (−1.088 kcal/mol).

Computer‐aided experiments are considered the most prominent initiatives for exploring novel lead compounds because not only do they save the time that would have been spent running a clinical trial, but also, most importantly, they save money (Singh et al., 2003). The SwissADME study demonstrated that all the identified compounds follow Lipinski's and Veber rules except hexadecanoic acid, methyl ester; pentadecanoic acid, methyl ester; and 11‐octadecenoic acid, methyl ester. Following these two rules is a significant indicator of good oral bioavailability and safety (Lipinski et al., 1997; Veber et al., 2002).

Furthermore, to establish our pharmacological studies, we evaluated the compounds using computer‐aided online tools called PASS. This study predicts the pharmacological potentials of lead molecules as Pa and Pi (Adnan et al., 2020b). Pa and Pi values fluctuate between 0.000 and 1.000. A lead molecule is considered experimentally active if Pa >Pi. Pa >0.6 indicates high probability of pharmacological potential, and values of 0.5 <Pa <0.6 reflect significant pharmacological potentials. Pa <0.5 indicates less pharmacological activity, which may indicate the chance of finding a new compound (Goel et al., 2011; Khurana et al., 2011). Here, the promising Pa value indicates that identified compounds have a wide range of pharmacological activity and possible targets against specific receptors suggesting that the compounds could be a new lead compound for cancer treatment.

## 5 CONCLUSIONS

In this study, we found that the CHF of *A. ferruginea* has strong polyphenolic content compared with the other fractions. GC‐MS quantitative phytochemical screening revealed that CHF contains 10 compounds, including octadecanoic acid which has known cytotoxic activity. In the DPPH and hydroxyl radical‐scavenging assay, CHF showed similar activity when compared to the standards BHT and CA, respectively. CHF also showed strong dose‐dependent inhibition of HeLa cell proliferation. The antioxidants and anticancer activities are supported by the presence of bioactive phytoconstituents, particularly different forms of octadecanoic acid methyl esters. Moreover, computational studies of identified compounds demonstrated drug‐likeness, safety, toxicological properties, and possible pharmacological activity, along with higher binding affinity for various receptors in molecular‐docking analysis. Therefore, the obtained biological activity of *A. ferruginea* supports its use as a traditional medicine against different illnesses, and the plant can be considered a potential candidate for treating cancer.

## CONFLICT OF INTEREST

The authors declare that they have no conflict of interests. All the authors read and approved to submit the manuscript for this journal.

## Data Availability

The data that support the findings of this study are available on request from the corresponding author.
